# The Correlation Between Stress of Conscience and Burnout Among Health Care Personnel at an Acute Care Hospital in Southern Sweden: A Cross‐Sectional Study

**DOI:** 10.1111/scs.70175

**Published:** 2026-01-09

**Authors:** Mia Ekstrand, Anna Ekwall, Susann Porter

**Affiliations:** ^1^ Department of Care Science, Faculty Health and Society Malmö University Malmö Sweden; ^2^ University Hospital of Skåne Malmö and Lund Sweden; ^3^ Department of Health Sciences, Faculty of Medicine Lund University Lund Sweden

**Keywords:** burnout, health care personnel, job demands‐resources model, nurses, stress, stress of conscience

## Abstract

**Introduction:**

Turnover rates among health‐care personnel are rising, which could jeopardise patient safety and the quality of care. One contributing factor to the high turnover is the prevalence of mental distress. Stress of conscience among healthcare personnel has been shown to affect them negatively, and feeling that they cannot provide the care their patients need increases their stress levels. Therefore, an increased understanding of stress of conscience, its relation to burnout and its consequences for healthcare can improve hospital care for both patients and staff.

**Aim:**

This study aimed to investigate the correlation between stress of conscience and burnout among health care personnel at an acute care hospital in southern Sweden.

**Ethical Issues and Approval:**

This study was approved by the Swedish Ethical Review Authority and followed the guidelines of the Declaration of Helsinki.

**Methods:**

The study used a descriptive cross‐sectional design. A total of 167 healthcare personnel at an acute care hospital in southern Sweden completed a questionnaire based on the Stress of Conscience Instrument and Oldenburg Inventory Burnout Instrument.

**Results:**

There was a positive correlation between the stress of conscience and burnout (*p =* 0.01, Spearman's rank correlation coefficient = 0.559). The results showed a statistically significant correlation between ‘living situation’ and stress of conscience, showing higher stress of conscience in groups living alone than in those living with a partner. The analysis also revealed a statistical significance between ‘working schedule’ and burnout, particularly in the groups working daytime and working shifts.

**Conclusion:**

Stress of conscience is positively correlated with burnout among healthcare professionals. Shift workers were more likely to experience burnout, and HCP living alone reported higher levels of stress of conscience. These findings highlight the combined impact of personal and organisational factors, underscoring the need for interventions that address both domains to reduce burnout and support workforce wellbeing.

## 1 Introduction

The turnover rates among health care personnel (HCP) have increased in many countries. Following the COVID‐19 pandemic, the increased work pressure led to even higher turnover rates among frontline HCP working in hospitals [1, 2], especially physicians, nurses and midwives [3]. One major problem for HCP that contributes to the high turnover is mental distress, and the workplace environment plays a role in this issue. Hospital work environments face challenges such as limited resources and increasing job demands, which impact the quality of care and work satisfaction among nurses [4]. To retain and attract staff, hospitals require organisational models that improve the workplace environment and support HCP's well‐being. One such model is implemented in ‘Magnet hospitals,’ which are hospitals that use institution‐wide interventions to promote and generate a positive work environment for health workers [2, 5]. The term ‘Magnet’ was developed in the United States in the 1980s to describe hospitals that attracted and retained nurses more than others, regardless of the national shortage. Research has shown that Magnet hospitals have common features that improve the quality of nursing. Specifically, they are characterised by five components: ‘structural empowerment; exemplary professional practice; transformational leadership; knowledge, innovations and improvements; and empirical outcomes’ [6].

Nurses are the largest group within HCP in almost all EU countries [3], and research shows that they leave their positions due to lack of work satisfaction [7, 8, 9]. The high nurse turnover rate has contributed to nursing shortage [10], which poses a risk for patient safety and can reduce the quality of patient care due to less patient contact time, poor teamwork, and a limited number of experienced personnel. For patients, the shortage of nurses leads to increased hospital‐acquired adverse events, fall rates, missed care, pressure ulcers [11] and higher mortality [12]. Previous research on the Magnet model has demonstrated that a good work environment, better nurse staffing and nurse involvement in decision‐making promote better patient outcomes, such as mortality and patient satisfaction [13]. In particular, participating in organisational decisions and being a part of shared governance protected nurses from emotional exhaustion, increased their work satisfaction and decreased their intention to leave their positions within a year [14]. Shared governance is fundamental to the Magnet model in terms of structural empowerment, implying that frontline nurses hold positions on organisational levels with the power to influence decisions that impact patient care [15].

In 2020, the Magnet4Europe project started with the aim of improving mental health and well‐being among HCP in Europe through organisational redesign. Six countries participated, including Sweden, which participated with four hospitals [2]. Early results demonstrated that a better work environment and staffing had positive correlations with lower reported adverse health indicators, care quality and patient safety [5]. As part of this initiative, a research project was started at an acute care hospital in southern Sweden to investigate the correlation between stress of conscience and burnout; this current study is a part of the research project.

### 1.1 Work Environment

The definition of a work environment includes its organisational environment—namely its policies and conditions, such as its structure, management, requirements, resources and responsibilities. It also includes the social environment, including how well staff work together, how much control they have over their work situation and what opportunities they have for professional development. The physical work environment consists of ergonomic factors such as furniture, space, ventilation and noise [16]. The hospital environment is a place with exposure to stressors, such as high job demands, lack of time and shortage of both material and human resources. Therefore, strategies for improving HCP work environments target work anxiety, stress, depression and burnout [17]. The better the work environment, the less likely it is to experience emotional exhaustion and burnout [18, 19, 20].

### 1.2 Burnout

The term ‘burnout’ was first used in a clinical sense in the United States in the 1970s to refer to a phenomenon in healthcare contexts. Its most influential definition was developed by Christina Maslach in the 1980s [21], who characterised it as ‘a syndrome of emotional exhaustion, depersonalisation and reduced personal accomplishment’ [22]. In effect, burnout is an extended reaction to chronic organisational stress. It is a worldwide health problem in the workplace, but Sweden is currently the only nation that recognises ‘burnout syndrome’ as a formal medical diagnosis. However, it will soon remove it as a diagnosis in line with the WHO's latest version of the International Classification of Diseases (ICD‐11) to apply a more universal classification system [23].

Stress‐related diagnoses and other common mental disorders are the most common reason for extended sick leave among female HCP in Sweden [24]. Both work‐related and personal stress have demonstrated a positive correlation with burnout. In particular, the symptoms of burnout are strongly correlated with a lack of organisational support and job demands [25]. Other burnout predictors include low nurse staffing levels, time pressure, low schedule flexibility and psychological demands. The consequences of burnout among HCP are reduced job performance, poor patient safety, higher infection rates, lower quality of nursing care and negative experiences of the care given among patients [19, 26]. Research has demonstrated that an intervention in the form of higher education, such as master's or doctoral degrees, enhances nurses' career success, helping to decrease burnout, reduce the levels of stress of conscience, improve the work environment and develop competency [27]. Burnout could be avoided if the social work environment had adequate resources such as teamwork and collaboration [28]. Additionally, support from family and community could alleviate the experience of work‐related stress or stress of conscience [25].

### 1.3 Stress of Conscience

Stress of conscience occurs when there is a dissonance between the care an employee wants to give and the care they can give, as well as between the person and the society and between the moral values and the actions taken [29, 30]. Other factors contributing to stress of conscience among HCP are having to manage different demands from patients and other staff, being unable to relieve suffering or having to provide care that ‘feels wrong’ [31]. Moreover, HCP such as physicians and nurses have been reporting an increasing amount of emotional distress, which is influenced by the work environment [2]. In this context, ‘emotional distress’ refers to the huge responsibility that leads to fear of failure. Work environments that create such distress often cause emotional exhaustion and depersonalisation [18, 21, 32]. These two dimensions of burnout have a positive correlation with stress of conscience and are also factors that contribute to nurses' intention to leave [18, 31].

HCP who report a higher level of stress of conscience also report more health complaints, such as sleep problems [31, 33]. When HCP do not live up to their own standards of delivering care, they experience a burdened conscience and, consequently, suffer from stress of conscience [34]. Stress of conscience also increases due to factors such as lack of time to provide care or listen to the patients, lack of collegial support and high workload [35]. In contrast, low levels of stress of conscience are associated with organisational and environmental support [33]. One factor for lower levels of stress of conscience and less intent to leave among HCP is whether they perceive their workplace or the care they provide to be person‐centred [36, 37]. Person‐centred care includes creating a partnership with the patients and seeing the person behind the diagnoses, taking part in the narratives and documenting and creating a plan through shared decision‐making with the patient [38].

The job demands‐resources (JD‐R) model is commonly used to predict well‐being, engagement and outcomes at both the individual and organisational levels in nursing practice [39]. The model categorises the factors associated with work stress into two categories: job demands and job resources. Job demands refer to the physical, psychological, social or organisational work aspects that require physical or mental efforts to handle. On the other hand, job resources are the work aspects that help reach work‐related goals, decrease work demands and stimulate development and learning [40]. The balance between these factors is crucial to prevent burnout and to promote well‐being [11, 35]. According to the JD‐R model, job demands such as time pressure and emotionally demanding contact with patients are strongly connected to feelings of exhaustion [41]; in contrast, the job resources autonomy at work and social support from managers and colleagues are connected to engagement and job satisfaction [40]. Consequently, the risk of getting affected by stress and burnout increases when job demands are high and job resources are low.

Stress of conscience and burnout are factors that may contribute to HCP leaving healthcare, thereby creating a shortage of staff. Therefore, investigating the correlation between these factors can facilitate creating a positive work environment and enhancing healthcare [5]. Additional factors that may underlie burnout include personal and organisational aspects; therefore, healthcare professionals' living situation is also relevant [19, 25, 26]. By examining these factors from a JD‐R model perspective, we can continue to develop strategies to improve the quality of care for patients. Examining these dimensions through the JD‐R model offers a valuable framework for developing targeted interventions that reduce burnout, strengthen staff wellbeing and ultimately improve the quality of patient care. In this context, job resources such as adequate staffing and supportive leadership can buffer the impact of high job demands, mitigating stress and preventing burnout.

### 1.4 Aim

This study aimed to investigate the correlation between stress of conscience and burnout among health care personnel at an acute care hospital in southern Sweden. It was further hypothesised that there would be a difference between stress of conscience, burnout and characteristics.

### 1.5 Hypothesis

We hypothesised that there would be a positive correlation between stress of conscience and burnout among health care personnel at an acute care hospital in southern Sweden. We also hypothesised that there would be a difference in the correlation between stress of conscience and burnout based on profession, years in the profession, years at the current workplace, work schedule and living situation.

## 2 Method

### 2.1 Design

This study had a cross‐sectional design. A cross‐sectional design is appropriate for describing the status of a phenomenon or correlation at a specific point in time. In the positivist paradigm, the underlying factors and correlations of phenomena are examined to ensure an objective and empirical understanding. This approach emphasises gathering and analysing empirical evidence and strives for generalisability [42]. This study was part of a larger project evaluating stress of conscience, work satisfaction and burnout among HCP in acute care units at a hospital in southern Sweden.

### 2.2 Participants

Eligible participants were nurse assistants, registered nurses, specialist nurses, physiotherapists and physicians who worked bedside in all departments of inpatient care. These are the most common occupations in inpatient care in hospitals [13]. No limitation was set regarding years in the profession and years in the current position (i.e., at the current workplace).

### 2.3 Data Collection

Data was collected between January and May 2024 through a questionnaire using the web application REDCap (REDCap, [43]). Information about the study, its aims, and the questionnaire was e‐mailed to all area managers and unit managers of departments at an acute care hospital in the south of Sweden, with a request to send it to all their employees according to the inclusion criteria. The e‐mail also included a QR code to scan to participate in the survey. REDCap was used to design the questionnaire and send and collect data. The questionnaire included two instruments: the Oldenburg Burnout Inventory [44, 45] and the Stress of Conscience Questionnaire [31]. It also collected demographic data with questions regarding profession, years in profession, years at current workplace, work schedule and living situation; the exact wording of demographics can be viewed in Table 1. In total, the questionnaire consisted of 88 questions.

**TABLE 1:** Demographic characteristics of the study participants.

Characteristics	*n* = 167	%	Mean (SD)
Profession			
Nurse assistants	55	32.9	
Physicians	10	6.0	
Physiotherapists	0	0	
Registered nurses	14	8.4	
Specialist nurse	60	35.9	
Other	10	6.0	
Missing	18	10.8	
Years in profession			
0–6	15	9	16.4 (10.38)
7–12	14	8.8	
13–18	12	7.2	
≥ 19	25	15	
Missing	101	60	
Years at current workplace			
0–6	71	42.5	7.72 (7.04)
7–12	39	23.3	
13–18	17	10.2	
≥ 19	8	4.8	
Missing	32	19.2	
Working schedule			
Day	98	58.7	
Night	12	7.2	
Shift	55	32.9	
Missing	2	1.2	
Living situation			
Single	39	23.4	
Single with children	19	11.4	
Partner	42	25.1	
Partner with children	64	38.3	
Missing	3	1.8	

### 2.4 The Oldenburg Burnout Inventory

The Oldenburg Burnout Inventory (OLBI) consists of 16 questions and measures burnout levels. The OLBI assesses the two dimensions of burnout—exhaustion and disengagement—with eight questions for each dimension. The eight questions comprise four negatively worded and four positively worded questions, and in the analysis, the positively worded questions were reversed. The responses range from 1 to 4, where 1 = totally disagree and 4 = totally agree [46]. The sum score ranges from 16 to 64, where a score of 44–59 indicates a moderate burnout level and > 59 indicates a high one [47]. The OLBI has been tested for construct and factorial validity [48]. Previous studies have reported a Cronbach's alpha of 0.83 for both dimensions assessed in the OLBI [49].

### 2.5 The Stress of Conscience Questionnaire

The Stress of Conscience Questionnaire (SCQ) consists of nine questions, each consisting of two parts: A and B. The A part describes situations in patient care that can give the respondents a feeling of ethical conflicts or stress of conscience answered on a scale ranging from *never* to *every day*. The B part uses a visual analogue scale ranging from 0 to 10, where 0 is ‘no, not at all’ and 10 is ‘yes, it gives me a strong feeling of stress of conscience’ [50]. The A score is multiplied by the B score to determine the total stress of conscience for each question. The total score for each SCQ question ranges 0–25, and the total SCQ score ranges 0–225 [51, 52, 53]. The SCQ was tested for reliability with Cronbach's alpha (0.83), and face and content validity were established in a Swedish context [50].

### 2.6 Data Analysis

Analyses were conducted both individually and collectively. Initially, a comprehensive analysis was performed, followed by partial analyses on subgroups, including separate examinations of individual professional categories. The collected data were exported from REDCap and were compiled and processed statistically with IBM SPSS (Statistics, Statistical Package for the Social Sciences version 29) [54, 55]. The data analysis was conducted in collaboration with a statistician from Lund University. The analysis of variance (ANOVA) test was used to analyse the data between the instruments, the mean scores and the demographic variables. One‐way ANOVA was used on the normal distribution data, testing the difference between the independent variables stress of conscience and burnout based on profession, years in the profession, years at the current workplace, work schedule and living situation. Tukey's post hoc test was used to assess the significance of the differences. The analysis was conducted on the total sample. The coefficients for the effect size measure eta squared (*η* ^2^) were calculated for non‐parametric data and interpreted using eta squared values: 0.01 for small, 0.06 for medium and 0.14 for large sample size [56]. Spearman's rank correlation was used to test the correlations between the results of the SCQ and OLBI. This test is appropriate for ordinal data [42]. Spearman's rank correlation coefficients were 0.0–0.30 for negligible, 0.30–0.50 for low positive, 0.50–0.70 for moderate, 0.70–0.90 for high positive and 0.90–1.00 for very high positive correlation [57]. The *p‐*values were calculated based on the corresponding null hypotheses: if a *p*‐value was less than or equal to 0.05, the null hypothesis was rejected, and the difference was considered significant.

### 2.7 Ethical Considerations

According to the declaration of Helsinki, the requirements that must be fulfilled are information, consent, confidentiality and utilisation [58]. In addition to receiving information about the study, the participants were informed of the following: Filling out the questionnaire was considered as consent for participation, and the participants could stop their participation at any time. For confidentiality, no name, age, sex or personal identification number were collected. Ethical approval was obtained from Swedish Ethical Review Authority (2023‐03158‐01).

## 3 Results

A significant positive correlation was found between stress of conscience and burnout, leading to rejection of the null hypothesis. The study included 167 participants: 55 were nurse assistants, 10 were physicians, 14 were registered nurses and 60 were specialist nurses. No physiotherapist participated in the study, for unknown reasons. The mean length of time the participants worked in their profession was 16.4 years, and the mean length of time in their current workplace was 7.72 years. Most participants worked during the daytime (*n* = 98, 58.7%) followed by shift time (*n* = 55, 32.9%). There were 64 participants living with both a partner and children. The participants' characteristics are presented in Table 1.

### 3.1 Correlations Between Stress of Conscience and Burnout

The analysis revealed that a higher SCQ score among the participants was significantly correlated with a higher OLBI score (*p* = 0.01, Spearman's rank correlation coefficient = 0.559). Among the participants, this indicates that individuals experiencing greater stress of conscience also tended to report higher levels of burnout, reflecting a moderate positive rank correlation between these constructs.

### 3.2 Correlation Between Demographics, SCQ and OLBI

There was a statistically significant difference between the participants' living situation and SCQ for the groups ‘living single’ and ‘living with a partner.’ In particular, the results showed statistical significance between ‘living single’ and SCQ (*p* = 0.036, *η* ^2^ = 0.077), indicating that participants living single experience a higher stress of conscience. Furthermore, a statistical significance was found between ‘working schedule’ and OLBI for the groups working ‘day’ and ‘shift,’ where the shift workers showed the highest risk of burnout (*p* = 0.017, *η* ^2^ = 0.051). The demographic variables of profession, years in the profession and years at current workplace showed no statistically significant effect on stress of conscience and burnout when analysed using ANOVA and effect size (as shown in Table 2).

**TABLE 2:** ANOVA results for OLBI and SCQ scores across demographic and work‐related factors.

	OLBI		95% CI	SCQ		95% CI
	*p* value	*η* ^2^		*p* value	*η* ^2^	
Years at current workplace	0.093	0.049	(0.000, 0.120)	0.216	0.051	(0.000, 0.137)
Profession	0.785	0.012	(0.000, 0.039)	0.263	0.054	(0.000, 0.126)
Years in profession	0.065	0.111	(0.001, 0.124)	0.369	0.079	(0.000, 0.241)
Working schedule	0.017*	0.051	(0.000, 0.137)	0.230	0.027	(0.000, 0.100)
Living situation	0.665	0.010	(0.000, 0.043)	0.036*	0.077	(0.000, 0.168)

*Note:* Effect size eta squared (*η* ^2^) 0.01 for small, 0.06 for medium and 0.14 for large.

* *p* ≤ 0.05.

The results demonstrated a statistically significant correlation between stress of conscience and burnout, leading to the rejection of the null hypothesis. To enhance the robustness of these findings and reduce the likelihood of a Type II error, effect sizes were calculated. These effect sizes indicated a moderate to strong association between stress of conscience and burnout, as confirmed by both Spearman's rank correlation and ANOVA analyses [42]. This relationship suggests that stress of conscience is not merely an isolated phenomenon but a critical contributor to occupational strain. Consequently, these findings underscore the importance of implementing targeted strategies that address ethical stressors and workload demands, thereby promoting the psychological well‐being and long‐term sustainability of healthcare personnel.

## 4 Discussion

This study aimed to investigate the correlation between stress of conscience and burnout among HCP at an acute care hospital in southern Sweden. The findings indicate that the higher the stress of conscience experienced by participants, the more likely they were to suffer from burnout. Thus, the hypothesis that there is a correlation between stress of conscience and burnout has been confirmed. This is in line with previous studies on registered nurses and nurse assistants working in residential care facilities [53, 59, 60] and specialist nurses working in intensive care units [61].

The present study demonstrated a statistically significant correlation between working schedule day/shift and burnout, with shift workers being the most affected by burnout. This is in line with previous research showing that working shifts are associated with burnout, decreased job performance, higher rates of errors and more sleep disturbance, leading to patient care errors [62, 63]. Although adequate staffing is a part of job resources [64], from a preventive perspective, it is better to decrease job demands than to increase job resources [64]. On the other hand, the Magnet4Europe study stated that both nurses and physicians prioritised interventions for reducing burnout and increasing well‐being, with nurse staffing being the top priority [5]. Investing in continuous professional development among HCP also increases the quality of care, patient safety, job satisfaction and career [6, 19].

The results also found a significant correlation between HCP's living situation and stress of conscience. In particular, individuals living with children, regardless of whether they have a partner, experience a lower degree of stress of conscience. This finding aligns with previous studies suggesting that being married and having children is protective against burnout [65]. Although burnout levels did not demonstrate statistically significant differences based on living situation in this study, previous research among HCP has reported such differences. This discrepancy highlights the complexity of burnout and suggests that the living situation may still play a role in burnout for some individuals. This is also evident in the responses to a single question regarding work influencing private life, which had the highest mean score and is consistent with several other studies [51, 52, 60, 66, 67]. The JD‐R model emphasises the need for balance between job resources and job demands, indicating that a lack of job resources and high job demands will increase the risk for stress and burnout [41].

Interestingly, although the present study did not directly ask for the participants' age but collected years of experience instead, the results did not demonstrate any correlation between years in occupation, years at current workplace and higher degrees of stress of conscience or burnout. This contradicts previous studies that identified older age, albeit not years in occupation, as a risk factor of stress of conscience. These results could be attributed to the fact that younger participants and participants with less experience were more likely to experience stress of conscience [65, 66].

Furthermore, the present study's results found no significant correlation between profession and stress of conscience or burnout. This was also observed in a review by Boucher et al., who found little variation in outcomes across professions regarding mental health problems such as burnout and perceived stress [1] and no differences between registered nurses and nurse assistants regarding stress of conscience [20, 68]. In contrast, results from various European healthcare systems report that job demands are high overall and twice as high among nurses compared to all healthcare personnel [3]. These results underscore the importance of understanding how stress and burnout can affect HCP, regardless of their specific roles.

The balance in the JDR‐ model, between job resources and job demands is a crucial factor to understanding work‐related stress and its consequences. Time constraints in healthcare settings have been widely documented in previous research in home‐ and psychiatric care and in hospital settings [34, 51, 60, 66, 67, 69], and a lack of time to deliver adequate patient care, leading to missed care, is correlated with burnout and lower quality of care and patient safety [70]. Spending quality time with patients could positively impact stress of conscience and lead to a clear conscience [66, 71]. Both more time and contact with patients and professional development are a part of the job resources that lead to better work balance [40]. Staffing and professional development are a part of structural empowerment in the Magnet model, and these components contribute to increased satisfaction among nurses and patients. According to Aiken et al. [5], HCP prioritised interventions that involved reducing documentation and implementing organisational changes to support HCP in spending more time with their patients [5]. Another intervention in staffing and resources could include increasing the nurse‐to‐patient ratio and improving retention and attraction [13, 70]. Transformational leadership, which involves engaging leaders who empower their staff and create a positive environment, is another relevant component of the Magnet model. An engaged leader enhances well‐being, as evidenced by increased work engagement and reduced burnout. This is consistent with the JD‐R model, where job demands negatively affect the relationship between engaged leaders and staff well‐being. In relation to the results of this study, an engaged leader could lower the stress of conscience by supporting HCP and offering more job resources to enhance their well‐being [72].

Understanding stress of conscience and the work stressors for HCP is necessary, not only to decrease the levels of stress of conscience but also to promote awareness and initiate discussions about moral concerns and to develop methods to reflect and discuss on both individual and theoretical levels. By reducing stress and burnout, the chances of improving nursing excellence increase; in turn, according to the Magnet model, this will enable hospitals to retain and attract staff [5].

### 4.1 Strengths and Limitations

The cross‐sectional study design has been criticised for its limitations in examining changes over time as the data is collected only at a single point. However, this design was appropriate for investigating whether stress of conscience precedes burnout and to demonstrate correlations [42].

The data collection method was another limitation. Nevertheless, using an online questionnaire facilitated data collection. For instance, online questionnaires are less costly and time consuming than interviews, which is an important consideration due to HCP's reported lack of time [42]. Additionally, the online questionnaire did not collect personal information such as social identity number, sex or age to maintain the participants' anonymity, in line with recommendations from the Swedish Ethical Review Authority. While there was no official written consent form for participation, scanning the QR code and responding to the questionnaire were considered consent to participate in the study, so HCP had to willingly and voluntarily respond to it on their own time. Moreover, the two instruments used in the questionnaire were both tested for reliability and validity [46, 49, 50, 67]. Cronbach's alpha for SCQ and that for OLBI, indicating high reliability [42].

This study has an unknown total population due to unknown number forwarded emails with the questionnaire, which can affect calculating an appropriate sample size. However, a similar study that included a similar sample size (161 participants) also found strong correlations between burnout and job demands, workload, unmet care goals and stress of conscience [52]. Further, this study has an uneven number of participants from the different professions, which could limit the external validity. Participants identified their profession in the questionnaire, but 10 respondents selected ‘other,’ which could include, for example, occupational therapists. The exclusion of this profession from the listed options could be a limitation, and future research could address this by requiring participants to provide their profession in free text. The professions represented in the survey reflect the size of the professions in total at the chosen hospital. There were no participants from the group of physiotherapists for an unknown reason, but previous research has demonstrated this group's high workload and occupational stress and its correlation to burnout [73].

## 5 Conclusions and Implications

The results indicate a significant positive correlation between stress of conscience and burnout among HCP, suggesting that these constructs are closely intertwined rather than isolated. Shift workers were more likely to experience burnout, and HCP living alone reported higher levels of stress of conscience. These findings emphasise the importance of both personal and organisational factors in understanding stress of conscience; addressing and managing situations that induce stress of conscience is important for enhancing the quality of health services. To further improve outcomes in line with the JD‐R model, hospital management should focus on increasing job resources while reducing job demands. Interventions aimed at burnout and stress of conscience should prioritise enhancing nurse resources, improving work environments and implementing organisational redesigns rather than focusing on individual interventions. The reason for the imbalance between the participating professions is unknown. A future study should be conducted through in‐depth interviews to gain a deeper understanding of stress of conscience among the different professions. Future research could also incorporate the patient's perspective on healthcare personnel's stress of conscience and burnout, in order to enhance patient safety and the quality of care.

## Author Contrubutions

**Mia Ekstrand:** data analysis, writing – original draft, writing – review and editing. **Anna Ekwall:** conceptualization, data curation, writing – review and editing. **Susann Porter:** visualisation, writing, review, editing and supervision. All authors reviewed the manuscript.

## Funding

The authors have nothing to report.

## Ethics Statement

The study was approved by the Swedish Ethical Review Authority (ref. number 2023‐03158‐01).

## Conflicts of Interest

The authors declare no conflicts of interest.

## Data Availability

Research data are not shared.
